# Man with Total Knee Arthroplasty Now Unable to Extend the Joint

**DOI:** 10.5811/cpcem.2018.2.36642

**Published:** 2018-03-14

**Authors:** Matthias Barden, Julie K. A. Kasarjian

**Affiliations:** Eisenhower Medical Center, Department of Emergency Medicine, Rancho Mirage, California

## CASE PRESENTATION

**Image 1:**
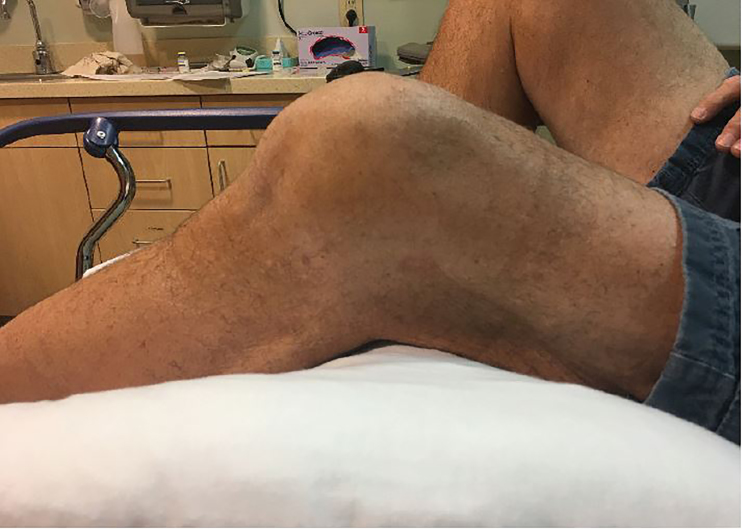
Photo of knee showing the position of locked flexion during a cam-jump dislocation of prosthetic knee.

**Image 2:**
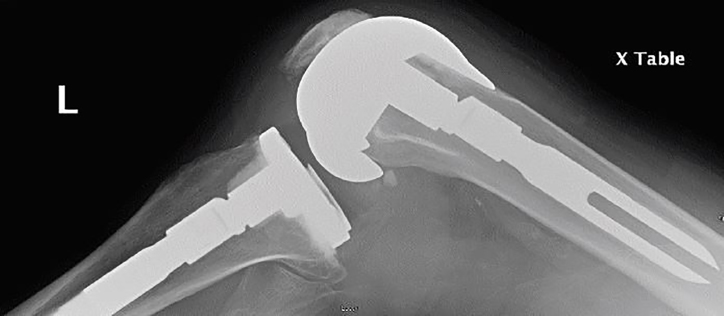
Initial lateral radiograph of cam-jump dislocation of prosthetic knee joint. The femoral component is subtly displaced anteriorly versus the tibial component of the prosthesis.

A 70-year-old man presented to the emergency department (ED) complaining of inability to extend his left knee. He had been kneeling on the ground pushing a heavy box when he felt a “pop” in the knee. Prior history included remote total knee arthroplasty complicated by instability requiring multiple revisions. On physical exam, the leg was distally neurovascularly intact and his knee was flexed at about 45° with limited range of motion (Image 1). Radiographs were performed; however, imaging was somewhat limited by his decreased mobility and pain (Image 2).

## DISCUSSION

**Diagnosis:** *Flexion (cam-jump) dislocation of total knee arthroplasty*

Instability of the knee joint after total knee arthroplasty is a somewhat common complication; however, dislocation is rare and represents the most severe degree of instability.^1^ The patient stated that he had “jumped the cam,” which was the term his orthopedist used when he had a similar episode two years previously.With this type of dislocation, hyperflexion of the joint causes the femoral component (“cam”) of the prosthesis to be rotationally translated anteriorly relative to the tibial component (“post”).^2^ The posterior aspect of the femoral component becomes locked in articulation with the tibial component and the patient is unable to extend the joint back into a normal position.

**Image 3:**
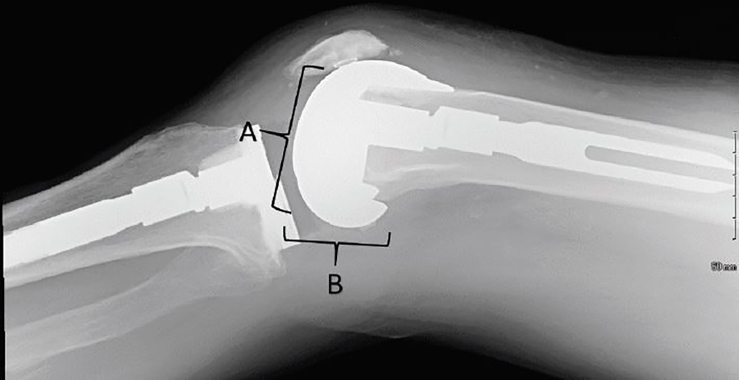
Post-reduction radiograph showing proper articulation of the prosthetic components. Prior to the reduction, the posterior aspect of the femoral component (B) articulated with the tibial component. After reduction, the inferior aspect of the femoral component (A) now articulates with the femoral component.

After discussing the case with the orthopedic surgeon, the patient underwent procedural sedation and reduction in the ED. Reduction was accomplished with hyperflexion of the knee to exaggerate the deformity, followed by extension to re-establish the normal articulating position of the prosthetic.^3^ Post-reduction radiographs were obtained showing normal articulation of the prosthesis components (Image 3). After reduction the patient was able to ambulate without any pain and had full range of motion. The orthopedic surgeon did not recommend splinting or a knee immobilizer, but was very clear that the patient should be given instructions to never kneel again to avoid this rare and painful complication.

**CPC-EM Capsule**

What do we already know about this clinical entity?

Orthopedic literature has described a translational or “cam-jump”-type of dislocation injury to total knee prosthetics, locking the knee in a semi-flexed position.

What is the major impact of the image(s)?

The images and discussion demonstrate the biomechanical mechanism by which the prosthetic becomes locked in this abnormal articulation.

How might this improve emergency medicine practice?

Understanding this mechanism will aid with diagnosis as well as reduction, which can likely be accomplished in the emergency department under procedural sedation.

Documented patient informed consent and/or Institutional Review Board approval has been obtained and filled for publication of this case report.

## References

[1] Sharkey PF (2002). Why are total knee arthroplasties failing today?. Clin Orthop Relat Res.

[2] Wang CJ, Wang HE (1997). Dislocation of total knee arthroplasty. A report of 6 cases with 2 patterns of instability. Acta Orthop Scand.

[3] Arumilli BR, Ferns B, Smith M (2009). Non-traumatic dislocation (cam jump) in a revision knee: A case report. Cases J.

